# An APOBEC3 Mutational Signature in the Genomes of Human-Infecting Orthopoxviruses

**DOI:** 10.1128/msphere.00062-23

**Published:** 2023-03-15

**Authors:** Diego Forni, Rachele Cagliani, Uberto Pozzoli, Manuela Sironi

**Affiliations:** a Bioinformatics, IRCCS E. Medea Scientific Institute, Bosisio Parini, Lecco, Italy; University of Michigan

**Keywords:** APOBEC, monkeypox, orthopoxvirus, smallpox, variola virus

## Abstract

The ongoing worldwide monkeypox outbreak is caused by viral lineages (globally referred to as hMPXV1) that are related to but distinct from clade IIb MPXV viruses transmitted within Nigeria. Analysis of the genetic differences has indicated that APOBEC-mediated editing might be responsible for the unexpectedly high number of mutations observed in hMPXV1 genomes. Here, using 1,624 publicly available hMPXV1 sequences, we analyzed the mutations that accrued between 2017 and the emergence of the current predominant variant (B.1), as well as those that that have been accumulating during the 2022 outbreak. We confirmed an overwhelming prevalence of C-to-T and G-to-A mutations, with a sequence context (5′-TC-3′) consistent with the preferences of several human APOBEC3 enzymes. We also found that mutations preferentially occur in highly expressed viral genes, although no transcriptional asymmetry was observed. A comparison of the mutation spectrum and context was also performed against the human-specific variola virus (VARV) and the zoonotic cowpox virus (CPXV), as well as fowlpox virus (FWPV). The results indicated that in VARV genomes, C-to-T and G-to-A changes were more common than the opposite substitutions, although the effect was less marked than for hMPXV1. Conversely, no preference toward C-to-T and G-to-A changes was observed in CPXV and FWPV. Consistently, the sequence context of C-to-T changes confirmed a preference for a T in the −1 position for VARV, but not for CPXV or FWPV. Overall, our results strongly support the view that, irrespective of the transmission route, orthopoxviruses infecting humans are edited by the host APOBEC3 enzymes.

**IMPORTANCE** Analysis of the viral lineages responsible for the 2022 monkeypox outbreak suggested that APOBEC enzymes are driving hMPXV1 evolution. Using 1,624 public sequences, we analyzed the mutations that accumulated between 2017 and the emergence of the predominant variant and those that characterize the last outbreak. We found that the mutation spectrum of hMPXV1 has been dominated by TC-to-TT and GA-to-AA changes, consistent with the editing activity of human APOBEC3 proteins. We also found that mutations preferentially affect highly expressed viral genes, possibly because transcription exposes single-stranded DNA (ssDNA), a target of APOBEC3 editing. Notably, analysis of the human-specific variola virus (VARV) and the zoonotic cowpox virus (CPXV) indicated that in VARV genomes, TC-to-TT and GA-to-AA changes are likewise extremely frequent. Conversely, no preference toward TC-to-TT and GA-to-AA changes is observed in CPXV. These results suggest that APOBEC3 proteins have an impact on the evolution of different human-infecting orthopoxviruses.

## INTRODUCTION

Mpox is an infectious disease caused by monkeypox virus (MPXV), a member of the genus *Orthopoxvirus*, which also includes variola virus (VARV; the causative agent of smallpox), vaccinia virus (VACV; used as a smallpox vaccine), and other zoonotic viruses, such as cowpox virus (CPXV). Until recently, mpox was considered a rare zoonotic disease occasionally transmitted in an area of endemicity that ranges from West to Central Africa. In the last 5 years, though, the prevalence of mpox has been increasing, both in Africa and worldwide (1). In particular, since the beginning of May 2022, a multicountry outbreak has caused more than 85,449 cases in 110 countries, with 98 deaths (as of 31 January 2023). The epidemiology of the ongoing outbreak is distinct from that previously observed in Africa: viral spread is sustained by human-to-human transmission, often mediated by sexual contact (1). Because of distinctive epidemiological and genomic characteristics, it was suggested that the virus causing the outbreak should renamed hMPXV1, a nomenclature we have adopted herein (2).

**FIG 1:**
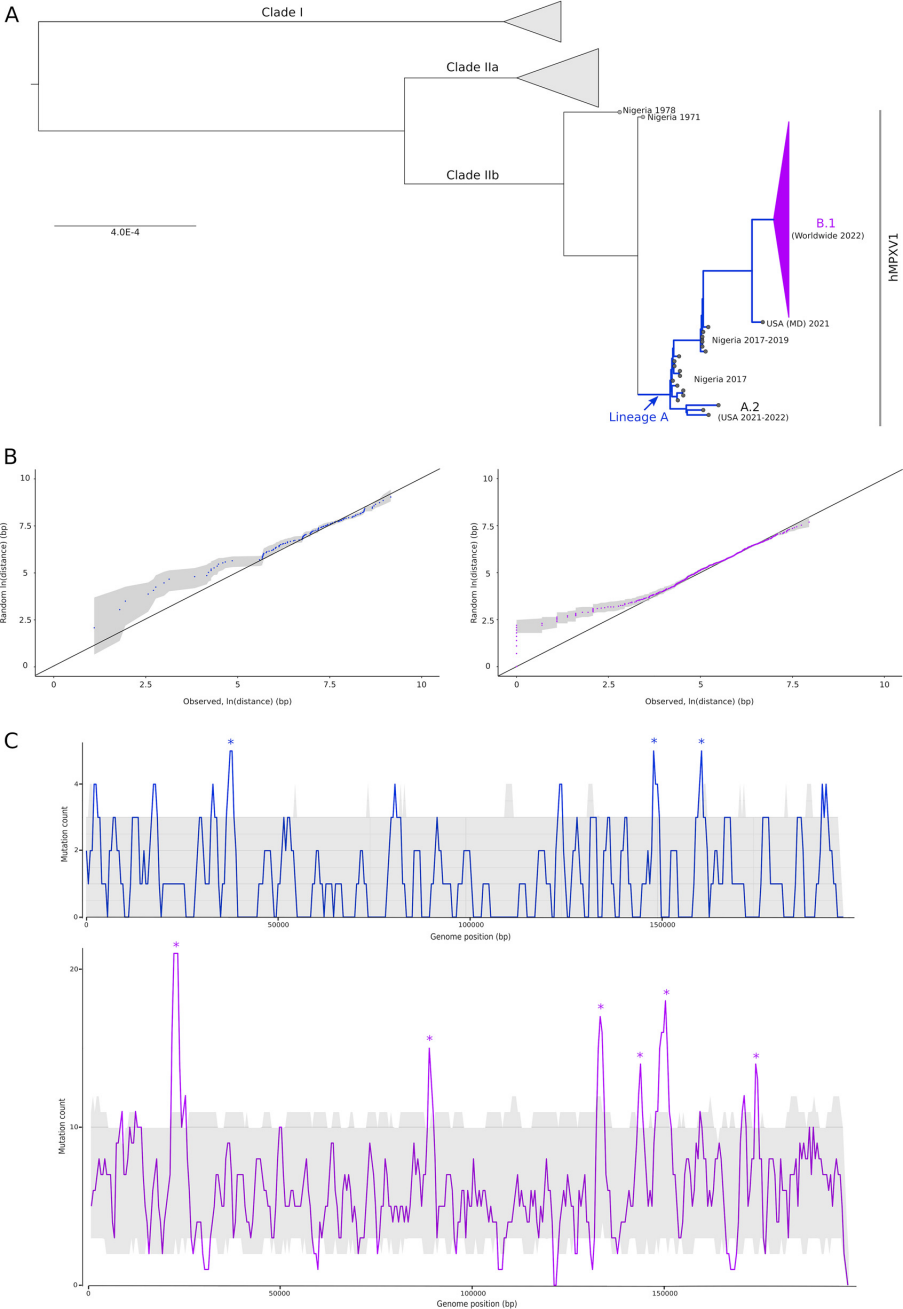
Clustering of mutations in hMPXV1 genomes. (A) Phylogenetic tree of 80 representative MPXV/hMPXV1 strains. A conserved genomic region (5) was used, and the tree was generated using IQ-TREE. Blue branches are the ones on which we counted mutations that accumulated in lineage A between 2017 and the emergence of the current predominant outbreak variant. The collapsed purple branches contributed mutations that have been accumulating during the 2022 outbreak. (B) Quantile-quantile plots of mutation distances in lineages A (left, blue) and B.1 (right, purple). Observed distances were plotted against the distances in 1,000 random genomes. Dots represent the medians; the gray shadows represent the 5th and 95th percentiles. (C) Sliding-window analysis of mutation counts. Mutations in lineages A (top, blue) and B.1 (bottom, purple) were counted in windows of 2,000 bp, moving with steps of 500 bp. The gray shadows represent the 5th and 95th percentiles of mutation counts in random genomes. Asterisks denote mutation hot spots.

Genomic surveillance of hMPXV1 showed that sequences sampled in 2022, as well as a few sampled in the United States in 2021, form a so-called lineage A. This lineage also includes a few Nigerian strains dating between 2017 and 2019 and is phylogenetically related to MPXV clade IIb. (Fig. 1A) (3, 4). Within lineage A, the predominant lineage B.1 accounted for the overwhelming majority of 2022 cases (Fig. 1A).

Lineages A and B.1 are characterized by several single-nucleotide substitutions. Most changes involve GA-to-AA or TC-to-TT replacements, leading to the suggestion that host apolipoprotein B mRNA editing catalytic polypeptide-like 3 (APOBEC3) enzymes have been driving the evolution of hMPXV1 since 2017 (3, 4, 6). If verified, this hypothesis would shed new light on the biology and evolution of poxviruses, which were previously thought to be resistant to APOBEC3-mediated restriction (7). Here, we used available hMPXV1 sequences, as well as VARV, CPXV, and fowlpox virus (FWPV) genomes, to show that the substitution spectra of orthopoxviruses during human transmission is dominated by the editing operated by APOBEC3 enzymes.

## RESULTS

### Genomic clustering of mutations.

The MPXV/hMPXV1 genome is an ~195 kb-long double-stranded DNA (dsDNA) molecule, with a GC content of about 33%. The mutations that characterize lineages A and B.1 tend to be distributed along the genome (3, 4, 6). However, APOBEC-generated mutations might be expected to cluster (8). We reasoned that some level of clustering was possibly missed because of the low GC content of the hMPXV1 genome and due to the relatively small number of mutations analyzed in previous studies. To determine whether this was the case, we retrieved hMPXV1 genomes available in public repositories, and we counted the number of mutations that accrued in lineage A between 2017 and the emergence of the current predominant outbreak variant B.1 (*n* = 121) (i.e., using strain Nigeria-SE-1971 as the reference), as well as mutations (*n* = 620) that have been accumulating during the 2022 outbreak (i.e., using strain MPXV_USA_2021_MD as the reference) (Fig. 1A). As expected, the majority of these mutations were G-to-A or C-to-T changes (83.5% in lineage A, 84.2% in lineage B.1). As an empirical comparison and to account for local differences in GC content, for both sets of mutations, we generated 1,000 mutated genomes by randomly changing the same overall number of C, T, G, and A nucleotides as in the real genomes. These are here referred to as “random genomes”. We next calculated the distance between consecutive mutations in real and random genomes. Quantile-quantile plots of distances in the real genomes against all 1,000 random genomes indicated that the observed mutations were significantly closer than the random ones (Fig. 1B).

**FIG 2:**
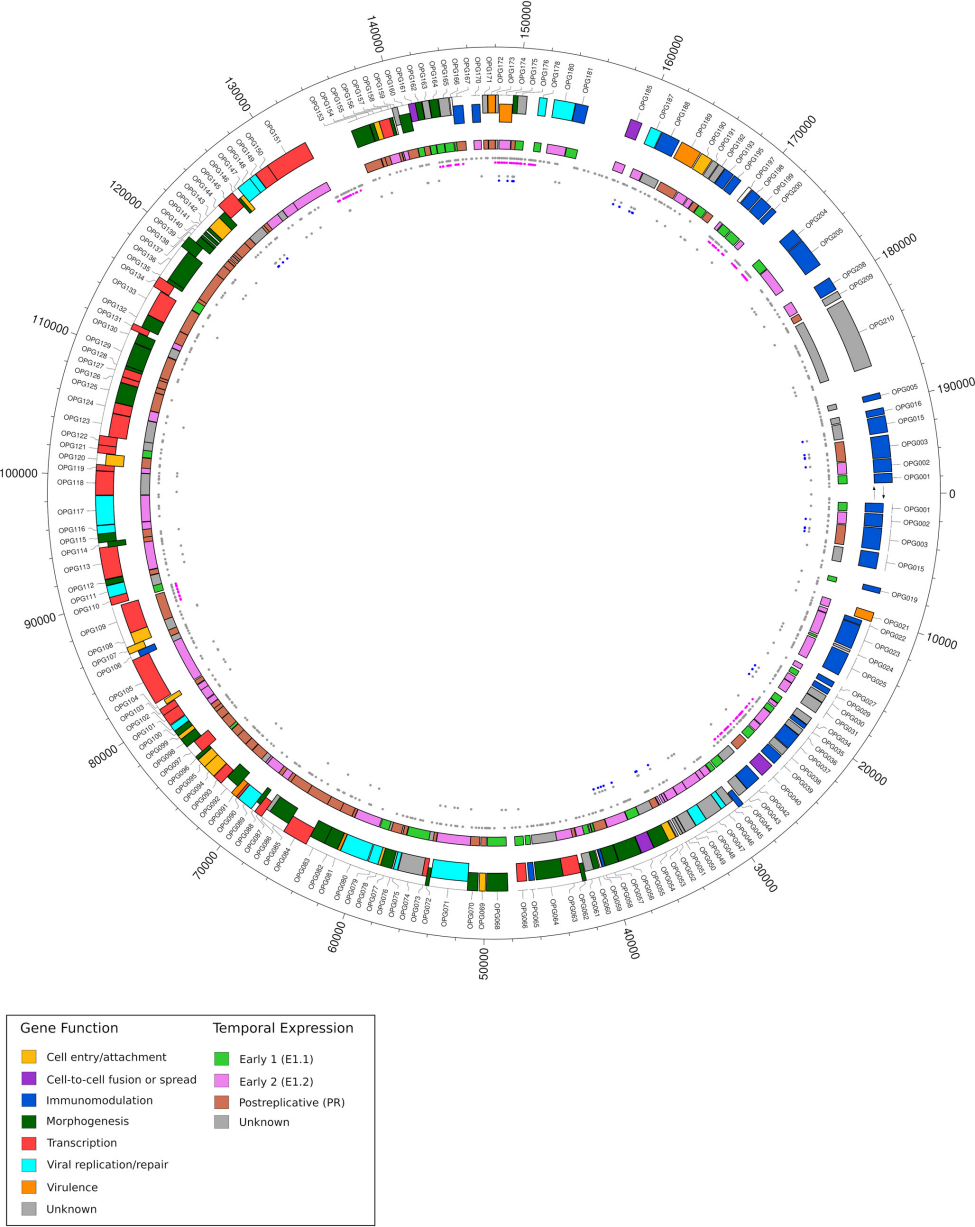
Context of mutations in hMPXV1 genomes. Circos plot of the hMPXV1 genome. The positions and annotation refer to strain MPXV-M5312_HM12_Rivers. Shown are (from the outside to the inside) genes color-coded according to function (1) and sense of transcription, genes color-coded according to temporal expression (9), all mutations (dark gray) and C-to-T/G-to-A mutations only (magenta) in lineage B.1, and all mutations (dark gray) and C-to-T/G-to-A mutations only (blue) in lineage A.

To determine which specific regions of hMPXV1 genomes represented mutation hot spots, if any, we counted the mutations in sliding windows. The same procedure was also performed for random genomes, and the 5th and 95th percentiles of mutation counts were calculated. Mutation hot spots were observed for both lineages A and B1, with limited overlap (Fig. 1C). This confirms the results obtained by calculating distances and indicates that substitutions are nonrandomly distributed and tend to cluster in specific regions. Mutation hot spots involve genes with diverse functions (Fig. 2).

### Positive association between expression levels and mutation occurrence.

**FIG 3:**
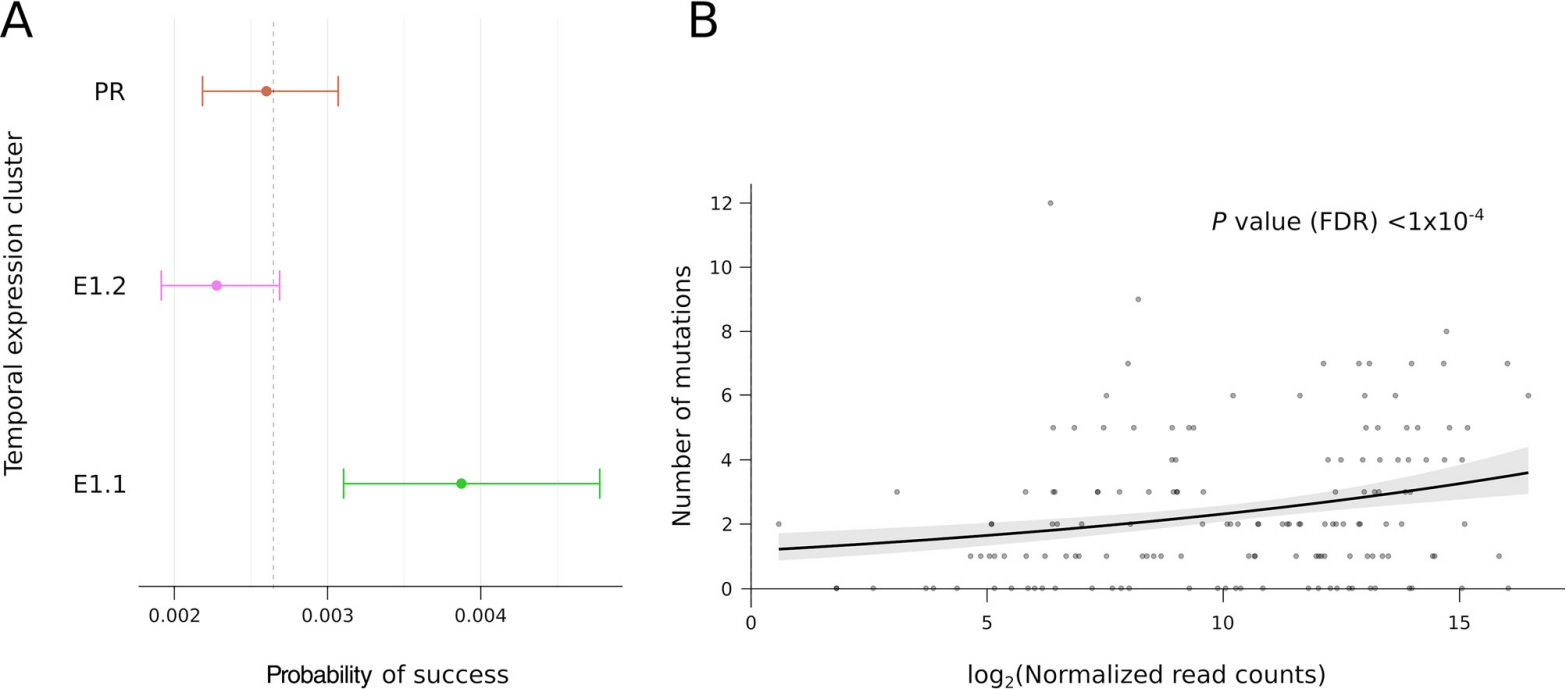
Correlation between mutation occurrence and gene expression. (A) Forest plot of the frequency of C-to-T/G-to-A mutations in genes from the three temporal expression clusters (early clusters, E1.1 and E1.2; postreplicative cluster, PR), indicated by solid circles, with whiskers representing the 95% confidence intervals, calculated using binomial tests. The dashed vertical line represents the expected probability of success (*P* = 0.00265). (B) Generalized linear model result of the relationship between C-to-T/G-to-A mutation counts and gene expression levels (read counts) at 2 h postinfection (gray dots). Gray shading indicates confidence intervals (9). The false discovery rate-corrected *P* value is also reported.

A previous study analyzed the temporal expression of viral genes during VACV infection (9). The authors grouped genes into two early (E1.1 and E1.2) clusters and a postreplicative (PR) cluster. We used these data to infer the expression timing of hMPXV1 genes, and we noted that mutation hot spots tended to be proportionally more common in early genes (Fig. 2). To formally test this association, we analyzed C-to-T and G-to-A mutations in lineage B.1 (because they are more numerous), and we performed binomial tests by taking into account the overall length of genes in each cluster. The results indicated that mutations were more common in E1.1 genes than expected by chance (Fig. 3A; binomial test, two-tailed, false discovery rate [FDR]-corrected *P* value = 0.0025).

Because E1.1 genes tend to be expressed at higher levels than E1.2 and PR genes (9), we assessed whether mutation occurrence correlated with gene expression levels (measured as read counts) at different times postinfection (0.5, 1, 2, and 4 h). Generalized linear models showed that at all time intervals, mutation occurrence increased with expression levels (Fig. 3B; see also Fig. S1 in the supplemental material). Overall, these results indicate that mutations preferentially affect highly expressed viral genes.

10.1128/msphere.00062-23.1 FIG S1 Generalized linear model result of the correlation between mutation counts and gene expression levels at different time points postinfection. False discovery rate-corrected *P* values are reported. At the 0.5-h time point, three overlapping points with very few reads represent outliers. Their removal from the analysis still yielded a significant result. Download FIG S1, PDF file, 0.07 MB. Copyright © 2023 Forni et al. 2023 Forni et al. https://creativecommons.org/licenses/by/4.0/ This content is distributed under the terms of the Creative Commons Attribution 4.0 International license.

### No transcriptional or replication asymmetry of C-to-T changes.

APOBEC3 proteins have been intensely investigated for their mutagenic role in human cancers. In cancer genomes, there is contrasting evidence for transcriptional asymmetry of APOBEC3-induced mutations (10–12). Replication asymmetry is instead observed, with most mutations occurring on the lagging strand (10–12). Conversely, APOBEC3 editing of human papillomavirus and of plasmid DNA instead occurs on both strands (13, 14).

We thus checked for asymmetries in C-to-T/A-to-G changes in hMPXV1 using mutations accumulating in lineage B.1. We observed very similar proportions of TC-to-TT mutations on both strands (51.6% on the plus strand and 48.4% on the minus strand). Assuming that, analogous to vaccinia virus (VACV), hMPXV1 has one single origin of replication at one end of the genome (15), this implies no replication asymmetry of mutations. Likewise, we found no evidence of transcriptional asymmetry (genes transcribed from the minus strand, 112 C-to-T changes and 99 G-to-A changes; genes transcribed from the plus strand, 105 C-to-T changes and 116 G-to-A changes). Thus, possibly because of distinct replication systems, the effects of APOBEC3 editing seem to differ depending on the target (genomic DNA or virus/plasmid).

### Context of C-to-T substitutions.

**FIG 4:**
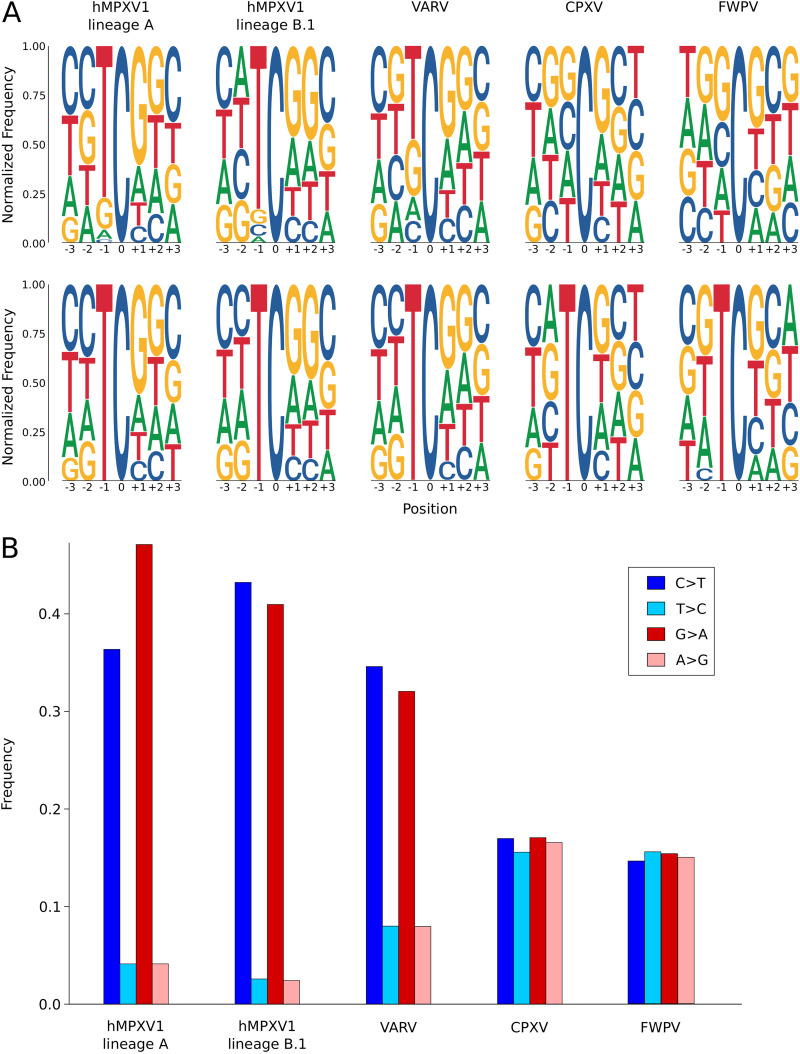
Mutation spectra in orthopoxviruses. (A) Top row, sequence context in which C-to-T mutations occur for hMPXV1, VARV, CPXV, and FWPV. The letter size represents the normalized frequency of each base flanking mutated cytidines. Bottom row, sequence context in which TC-to-TT mutations occur. (B) Frequencies of C-to-T, T-to-C, G-to-A, and A-to-G mutations in hMPXV1, VARV, CPXV, and FWPV are shown as a bar plot.

We next set out to analyze the sequence context of the observed substitutions. Because these are hypothesized to derive from the action of one or more APOBEC enzymes (i.e., from the deamination of cytosines), G-to-A mutations were considered C-to-T changes in the opposite strand. In addition, the counts of bases flanking the mutated cytosines were normalized by the frequency of each nucleotide in the hMPXV1 genome. For both lineages A and B.1, the results confirmed a very strong preference for a T in the −1 position (Fig. 4A). No marked preference was observed in the +1 position, although some slight overrepresentation (compared to the genome composition) of guanosines was evident.

Overall, these observations strongly support the view that the mutation process that is driving the accumulation of substitutions during ongoing human-to-human transmission was already operating in 2017, before the virus left Africa.

### Comparison with other poxviruses.

Finally, we compared the mutation spectrum of hMPXV1 to those of two other orthopoxviruses, VARV and CPXV. These were selected due to their different host ranges. VARV used to be a human-specific virus, and therefore, mutations in the viral genomes must result from processes that occurred in human cells. Conversely, CPXV is a zoonotic pathogen isolated from different mammals (including humans) but thought to be maintained and transmitted by rodents (16).

We retrieved 48 VARV and 24 CPXV genomes. In the case of VARV, we counted mutations that have accumulated in modern genomes using a Viking Age sequence as the reference (GenBank accession number LR800244). For CPXV, we selected viruses in clade 1 (because they are more numerous), and we used the oldest sample as the reference (KY463519; collection date, 1971).

To measure the asymmetry in the substitution spectrum of VARV and CPXV, as well as of hMPXV1, we counted the number of C-to-T and G-to-A mutations and of the opposite transitions (T-to-C and A-to-G). In VARV, C-to-T and G-to-A changes were extremely more common than the opposite substitutions, although the effect was slightly less marked than for hMPXV1 (Fig. 4B). Conversely, C-to-T and G-to-A changes in CPXV genomes had virtually the same prevalence as the opposite transitions (Fig. 4B).

As a further comparison, we analyzed 21 fowlpox virus (FWPV; genus *Avipoxvirus*) genomes. FWPV infects birds, which do not encode APOBEC3 protein (17). Again, the oldest sequence (GenBank accession number MW558073; dating to 1970) was used as the reference. As in the case of CPXV, C-to-T and G-to-A changes had a similar prevalence as the opposite substitutions in these genomes (Fig. 4B).

Analysis of the sequence context of C-to-T changes (as above, G-to-A changes were considered C-to-T substitutions in the opposite strand) confirmed a preference for a T in the −1 position for VARV, but not for CPXV or FWPV (Fig. 4A). Some preference for a G in the +1 position was observed in all cases.

To directly analyze the context of putative APOBEC3-mediated changes, we exclusively analyzed the context of TC-to-TT changes. For hMPXV1 and VARV, but not for CPXV and FWPV, we observed a slight preference for pyrimidines at the −2 position. The preference for a G in the +1 position was still present in all contexts but more evident for hMPXV1 and VARV (Fig. 4A).

## DISCUSSION

We investigated the mutation pattern of hMPXV1 with the aim of understanding the mechanism(s) responsible for the unusual accrual of mutations in circulating strains. Our working hypothesis was that human APOBEC3 proteins were the major determinants of mutation occurrence (3, 4, 6). We confirmed an overwhelming prevalence of C-to-T/G-to-A mutations, with a sequence context consistent with the preferences of several human APOBEC3 enzymes. We also found that mutations preferentially occurred in highly expressed genes. Overall, these observations lend strong support to the hypothesis that the mutation spectrum of MPXV/hMPXV1 has changed since 2017, and this is due to the editing activity of human APOBEC3 proteins.

As previously noted, a possible explanation for this twist in the evolution of hMPXV1 is that sustained circulation in the human population has exposed hMPXV1 to a different APOBEC3 repertoire than that of its natural host (most likely, African rodents) (3, 4). In fact, because of gene duplications followed by diversification, primates encode an expanded array of APOBEC3 enzymes compared to other mammals (17). To asses this possibility, we analyzed the mutation patterns of two other orthopoxviruses with distinct host ranges. The pattern of the human-specific variola virus was very similar to that of hMPXV1, in terms of both the mutation spectrum and the C-to-T sequence context. The less-evident skewing toward C-to-T/G-to-A changes and the lower preference for a T in the −1 position most likely derive from the fact that the substitution pattern of VARV has been shaped by the mutation process but also by the action of natural selection, acting over centuries of human transmission. In the case of the zoonotic CPXV, no skew toward C-to-T/G-to-A changes was observed, and no preferred sequence context was observed for C-to-T mutations. In this respect, the CPXV substitution pattern was very similar to that of FWPV, which infects birds. The CPXV genomes we included in the analyses were derived from different mammals (e.g., cats, alpacas, raccoons), which are, however, considered dead-end hosts, as the virus is thought have its reservoir in rodents (16). As a consequence, the bulk of sequence changes are expected to reflect the mutation/selection process in rodents, which encode only one APOBEC3 protein, with expression restricted to hematopoietic cells and lymphoid tissues (18). Because the mouse APOBEC3 protein displays variable sequence preferences even among laboratory strains (19), it is difficult to speculate about the possible preference in the CPXV reservoir, whose precise nature is still uncertain. Furthermore, mouse APOBEC3 has been mainly investigated in the context of retrovirus infection, where both deamination-dependent and -independent activities were shown to variably restrict different viruses (17, 19–21). It is thus possible that CPXV is restricted in rodent cells by deamination-independent mechanisms or that the bulk of APOBEC3-induced changes in hMPXV1 and VARV occurs in tissues/cell types that express one or more human APOBEC3 proteins, but where mouse APOBEC3 is not expressed. Thus, the reasons why CPXV is not edited by APOBEC3 in its natural host remain to be determined. However, the overall conclusion of our analyses is that orthopoxviruses transmitted in humans are edited by one or more host APOBEC3 enzymes.

As a corollary of this observation, it is worth noting that the ongoing mpox outbreak and historical smallpox epidemics were sustained by distinct routes of transmission: sexual for hMPXV1, through saliva droplets for VARV (22). Because the two viruses display a similar mutation spectrum, the pattern of transmission is unlikely to be a major determinant of the strength and frequency of APOBEC3-mediated editing. However, both smallpox and mpox are characterized by skin lesions, and it is possible that APOBEC3 proteins expressed in keratinocytes, fibroblasts, or other cells in the skin are responsible for editing (13, 23).

The observed sequence context of C-to-T mutations for hMPXV1 (and for VARV) may be consistent with the action of different APOBEC3 proteins which are expressed in the cytoplasm (3A, 3C, 3D, 3F, and 3H), where poxvirus replication occurs (8). Two recent studies reported a preference for pyrimidines at the −2 position in APOBEC3A-induced mutations (10, 24). A preference for a T (but not C) at the −2 position was also shown for APOBEC3F (25, 26). Also, for both APOBEC3A and APOBEC3F, a guanosine in the +1 position was reported to be overrepresented in some analyses (25, 27) but not others (10, 26). Most likely, APOBEC3 substrate specificities vary depending on the target (viral versus genomic DNA), the DNA secondary structure, and, possibly, other features (24, 28). Furthermore, although it was less marked than for hMPXV1 and VARV, we also observed an overrepresentation of a G in the +1 position of C-to-T changes for CPXV and FWPV. This implies that in all these viral genomes, a proportion of mutations occur at CpG sites, suggesting a role for the zinc finger antiviral protein (ZAP). The antiviral activity of ZAP is thought to drive the depletion of CpG dinucleotides observed in the genomes of several viruses that infect mammals and birds (29). It is thus possible that a small fraction of mutations in orthopoxvirus genomes are selected to deplete CpGs. Indeed, some poxviruses encode a ZAP antagonist (30), which may, however, fail to fully suppress the host protein, which is fast evolving in mammals and birds (29, 31).

The deamination activities of most APOBEC3 proteins (including 3F) have been mainly investigated in the context of HIV infection, whereas APOBEC3A has been intensely studied for its role as a mutagen in cancer genomes (8). However, APOBEC3A was also implicated in the editing and restriction of human papillomavirus (HPV), as well as in the deamination of foreign dsDNA (13, 14, 23). This enzyme is expressed in skin and mucosal tissues (13, 23). Conversely, APOBEC3F is mainly expressed in hematopoietic and lymphoid cells. Nonetheless, it is possible that hMPXV1 also infects skin-resident immune cells, where it may become a target of APOBEC3F deamination. Thus, identification of the enzyme responsible for introducing hMPXV1 mutations will be challenging due to the complex pattern of APOBEC3 expression *in vivo*.

Another open question is why hMPXV1 has accumulated a sizable number of mutations in a relatively short time, suggesting a poor efficiency for the repair of deaminated nucleotides. In the human genome, C-to-U lesions in DNA are repaired by a process initiated by uracil DNA glycosylase (UNG). UNG catalyzes uracil excision, resulting in an abasic site, which is repaired by cleavage, local DNA synthesis, and ligation (32). Human UNG is localized either to the nucleus (UNG2) or to the mitochondria (UNG1) and is therefore unavailable to poxviruses (32). These latter, however, encode their own UNG, which has an essential role in DNA replication, irrespective of its glycosylase activity (33). In fact, poxvirus UNG has distinctive sequence, structural, and functional features, and it is incorporated into the DNA polymerase complex, where it functions as a processivity factor (34). It was shown that uracil bases incorporated during DNA synthesis are excised by the catalytically active UNG in the polymerase complex (35). However, it is unclear whether free viral UNG can excise uracils that do not result from misincorporation during replication. Moreover, no enzyme with the ability to repair abasic sites has been identified in poxviruses to date (34). As a consequence, it is at present impossible to ascertain how poxviruses can repair APOBEC3-mediated deamination. Furthermore, because the extent of APOBEC3 editing is unknown, it is impossible to determine how often changes are repaired.

Whereas experimental analyses will be necessary to determine the source of APOBEC3-mediated editing in hMPXV1 genomes and the nature of repair mechanisms (or lack thereof), we found that mutations are more common in highly expressed genes. A similar observation was recently reported for some cancer genomes (11), explained by the fact that transcription leads to dsDNA unwinding, with the transient formation of single-stranded DNA (ssDNA) regions, a target of APOBEC3-mediated editing. The same authors also reported transcriptional asymmetry of APOBEC3 mutation signatures, a feature we do not observe in hMPXV1 genes. However, both the association with transcription levels and transcription asymmetry are still controversial findings in human cancer genomes (10–12, 36, 37). Thus, experimental analyses will be required to determine the molecular mechanisms of APOBEC3-mediated editing of hMPXV1 genomes and the role of transcription. In addition, a clear caveat of the gene expression analyses is that we inferred timing and transcript levels for hMPXV1 genes based on experimental data obtained in VACV infection. Although VACV and hMPXV1/MPXV share many biological features and have similar genomic architecture, specific experiments with hMPXV1/MPXV will benefit research on mpox.

In summary, our data strongly support a role for APOBEC3 enzymes in the editing of human-infecting orthopoxviruses. Using an approach slightly different from the one we applied herein, Poulain and colleagues recently reported that other human-infecting dsDNA viruses carry a footprint of APOBEC3-mediated selective pressure, detected as a depletion of APOBEC3-favored motifs (38). Specifically, they showed that papillomaviruses and polyomaviruses carry evidence of a strong selective pressure acting genomewide and on both strands. Conversely, in the case of gammaherpesviruses and adenoviruses, the footprint is localized to the lytic origins of replication. This is a clear indication that dsDNA viruses are targeted by APOBEC3s, although poxviruses differ from the viruses analyzed by Poulain in that they replicate in the cytosol (38). The notion that poxviruses are not targets of APOBEC3-mediated editing derives from the observation that APOBEC3G, 3F, or 3H have no effect on VACV replication (7), possibly because the poxvirus replication complex is sequestered in specialized regions of the cytoplasm known as “virus factories” (17). However, there are many possible reasons to explain the discrepancies between the findings in VACV infection and the evidence we present herein. For instance, editing of hMPXV1 may be operated by APOBEC3 enzymes other than 3G, 3F, or 3H. In this respect, 3A is a possible candidate, as it includes a single zinc-coordinating domain (17), and its smaller size (compared to that of 3G and 3F) might facilitate access to virus factories. Alternatively, VACV may be endowed with mechanisms to control APOBEC3 activity that are not functional in hMPXV1 and VARV. Kremer and coworkers showed that APOBEC3G is not degraded during VACV infection (7). However, distinct viruses have developed different strategies to control APOBEC3s. As an example, Epstein-Barr virus encodes the BORF2 protein, which inhibits APOBEC3B and relocalizes it to perinuclear bodies (i.e., away from the replication centers) (39). Finally, it is possible that APOBEC3 overexpression did induce some sublethal editing in VACV genomes that were not sequenced by Kremer and coworkers (7). Indeed, if APOBECs are responsible for mutations in hMPXV1 genomes, as we suggest, they are clearly insufficient to curb viral replication and spread. An interesting possibility is that APOBEC-induced mutations provide some fitness advantages to hMPXV1 during infection *in vivo*. This was previously suggested to be the case for HIV-1, as APOBEC3-induced changes can contribute to immune evasion, drug resistance, and transmissibility (17). Whether APOBEC3 proteins are providing some evolutionary advantage to hMPXV1, which is characterized by low mutation rates, remains to be evaluated. It is also possible, though, that slightly deleterious APOBEC3-driven mutations are accumulating in the hMPXV1 population, due to drift and bottlenecks at transmission. Continuing genomic surveillance of the outbreak is thus warranted by these data and by previous findings (3, 4, 6).

## MATERIALS AND METHODS

### Sequences, alignments, and phylogenetic tree.

hMPXV1 sequences were retrieved from the GISAID Initiative (https://www.gisaid.org) and from the National Center for Biotechnology Information (NCBI) databases (as of 20 October 2022). In particular, 1,603 complete, high-coverage, lineage B.1 genomes were retrieved from GISAID, and 21 complete/almost-complete lineage A genomes were downloaded from the NCBI nucleotide database. Strain lists are provided in Tables S1 and S2 in the supplemental material. We used as references strains Nigeria-SE-1971 (GenBank accession number KJ642617) and MPXV_USA_2021_MD (ON676708) for lineages A and B.1, respectively.

10.1128/msphere.00062-23.2 TABLE S1 List of hMPXV-1 GISAID IDs and originating laboratories responsible for obtaining the specimens. Download Table S1, PDF file, 0.06 MB. Copyright © 2023 Forni et al. 2023 Forni et al. https://creativecommons.org/licenses/by/4.0/ This content is distributed under the terms of the Creative Commons Attribution 4.0 International license.

10.1128/msphere.00062-23.3 TABLE S2 List of MPXV GenBank accession numbers. Download Table S2, DOCX file, 0.00 MB. Copyright © 2023 Forni et al. 2023 Forni et al. https://creativecommons.org/licenses/by/4.0/ This content is distributed under the terms of the Creative Commons Attribution 4.0 International license.

Complete VARV, CPXV lineage 1, and FWPV genome sequences were retrieved from the NCBI database. We used one Viking Age genome (GenBank accession number LR800244) and the oldest sample (KY463519) as the reference strains for the VARV and CPXV data sets, respectively. Likewise, for the FWPV analysis, we used the oldest sample (MW558073) as the reference strain. A list of analyzed genomes is reported in Table S3.

10.1128/msphere.00062-23.4 TABLE S3 List of VARV, CPXV, and FWPV strains. Download Table S3, DOCX file, 0.01 MB. Copyright © 2023 Forni et al. 2023 Forni et al. https://creativecommons.org/licenses/by/4.0/ This content is distributed under the terms of the Creative Commons Attribution 4.0 International license.

Whole-genome alignments for the five viral data sets and their corresponding references were generated using MAFFT v7.427 software (40) with default parameters. Substitutions were counted by comparing all positions of the alignment with the corresponding reference sequence; to exclude possible sequencing errors, we considered mismatches that occurred in at least two sequences. Nucleotide positions were then converted and referred to the reference genomes.

Eighty representative strains were selected to generate a MPXV/hMPXV1 phylogeny. The conserved genomic region (5) was aligned using MAFFT, and the tree was generated using IQ-TREE (41).

### Mutation distribution.

To compare hMPXV1 mutation distributions, we generated two sets of 1,000 random genomes using the two reference genomes as scaffolds. In particular, for each reference genome, we mutated the same number of nucleotides that we found mutated in lineages B.1 or A, by randomly choosing from all genomic positions of the same bases where mutations were observed (e.g., we observed 283 mutated cytosines in lineage B.1, and we mutated the same number of cytosines in each random genome). We then calculated the distance between consecutive mutations for each of the 1,000 random sequences. To assess whether the distances among the observed mutations were random, the observed distribution distances were compared with each of the 1,000 random distributions using a quantile-quantile plot.

The distribution of mutations was also analyzed along the hMPXV1 genome using sliding windows of 2,000 nucleotides and 500 nucleotide steps. We then counted the number of mutations falling within each window. Again, we performed the same analysis for the random genomes, and we compared the number of observed mutations with the distribution from random genomes within the same windows. To be conservative, we considered mutation hot spots to be only those windows in which the count of observed mutations was higher than the maximum value reached by the 95th percentile in random counts along the whole genome. All analyses were performed in the R environment (42).

### APOBEC mutation context.

C-to-T and G-to-A changes were analyzed in the context of APOBEC enzyme activity. Therefore, G-to-A changes were considered C-to-T changes occurring in the opposite strand, and the reverse complement sequences were analyzed. We retrieved +3 and −3 nucleotides flanking these mutations. Mutations falling in the inverted terminal repeats were counted only once to avoid redundancy. Nucleotide counts at each position were then divided by the overall nucleotide count in the genome and normalized for visualization clarity. Sequence logos were generated using the R package ggseqlogo (43).

### Gene expression levels and mutation profile.

Expression levels of vaccinia virus genes were retrieved from a previous study (9). We followed the authors’ classification, and we assigned each MPXV gene to one of the three temporal expression categories: two early (E1.1 and E1.2) clusters and a postreplicative (PR) cluster. Only clear MPXV orthologous genes to either the VACV-WR or VACV-Cop genes were assigned to temporal expression clusters. Read counts were retrieved from the same study and normalized as suggested by Yang et al. (9).

Binomial tests were run to assess whether each temporal expression cluster was enriched in mutated genes. For each cluster, we considered the number of successes as the number of G-to-A or C-to-T mutations in all genes in that cluster and the number of trials as the length of all genes in that cluster. The probability of success was set as the proportion of all G-to-A or C-to-T mutations in the whole reference genome. *P* values were then corrected for multiple testing using the FDR method.

The relationship between G-to-A and C-to-T changes and gene expression levels was analyzed using generalized linear models. For each time postinfection (0.5, 1, 2, and 4 h), we compared the count of mutations for each gene with the number of normalized reads for that gene. We used a Poisson distribution as the error distribution, and we corrected the generated *P* values using the FDR method.

All analyses were performed in the R environment.

### Data availability.

The data supporting the findings of this study are available from GISAID and NCBI. The accession numbers are listed in Tables S1 to S3.
