# Supplementary material for: An APOBEC3 Mutational Signature in the Genomes of Human-Infecting Orthopoxviruses
Source: mSphere. 2023 Mar 15;8(2):e00062-23. doi: 10.1128/msphere.00062-23 (PMC10117092; doi:10.1128/msphere.00062-23)

Supplementary Figure 1. Generalized linear model result of the correlation between mutation counts and gene expression levels at different time points post infection. False discovery rate corrected p-values are reported. At the 0.5h time point, three overlapping points with very few reads represent outliers. Their removal from the analysis still yielded a significant result.

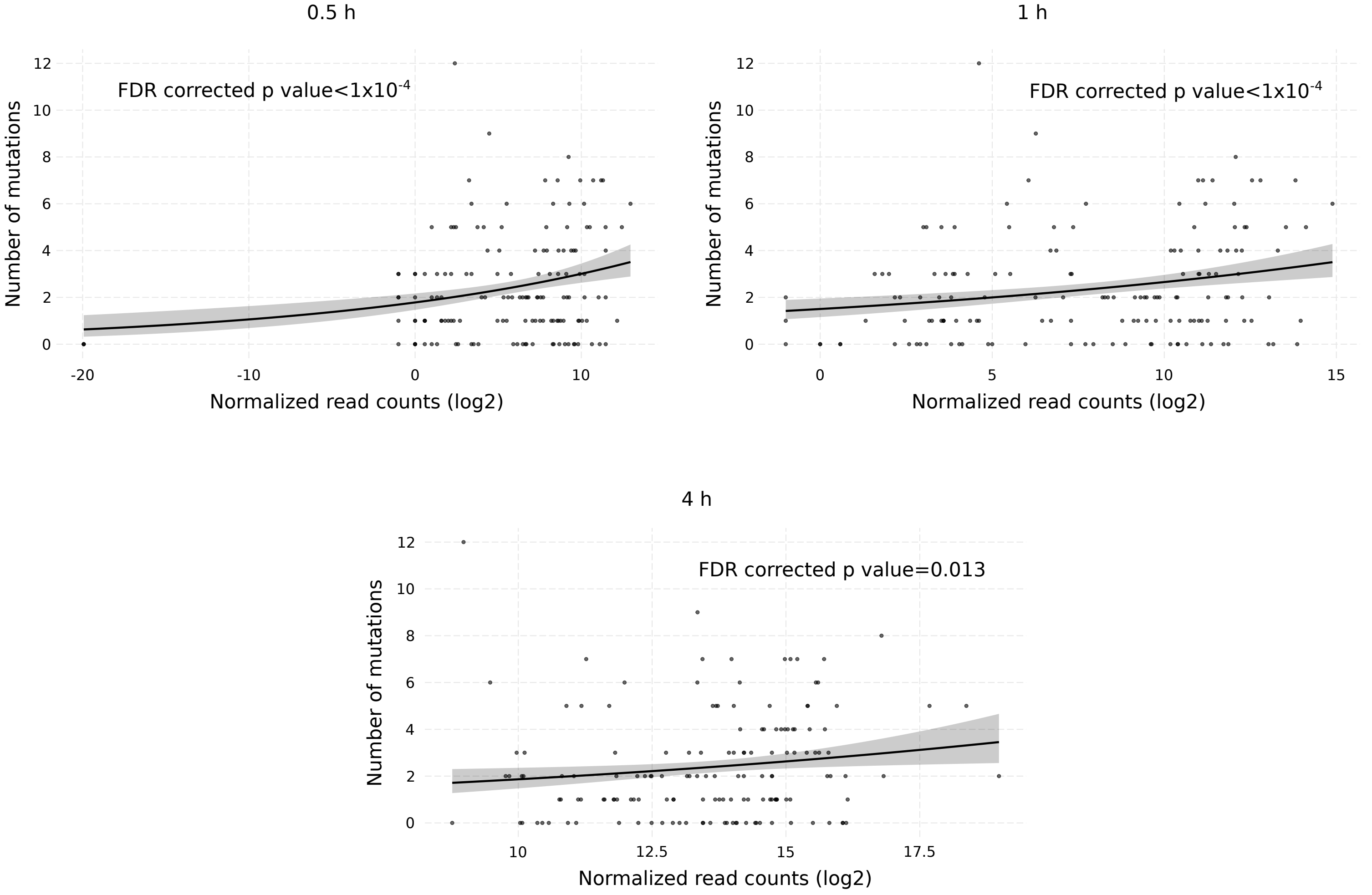

Supplement: FIG S1 [file msphere.00062-23-s0001.pdf]
