# Supplementary material for: An APOBEC3 Mutational Signature in the Genomes of Human-Infecting Orthopoxviruses
Source: mSphere. 2023 Mar 15;8(2):e00062-23. doi: 10.1128/msphere.00062-23 (PMC10117092; doi:10.1128/msphere.00062-23)
Supplement: TABLE S2 [file msphere.00062-23-s0003.docx]

**Supplementary Table 2. List of MPXV NCBI accession IDs.**

| **NCBI Accession ID** | **Strain Name** | **Host** | **Country** | **Collection Date** |
| --- | --- | --- | --- | --- |
| ON674051 | MPXV_USA_2022_FL001 | Homo sapiens | USA: FL | May-2022 |
| ON675438 | MPXV_USA_2022_VA001 | Homo sapiens | USA: VA | May-2022 |
| ON676707 | MPXV_USA_2021_TX | Homo sapiens | USA: TX | Jul-2021 |
| ON676708 | MPXV_USA_2021_MD | Homo sapiens | USA: MD | Nov- 2021 |
| MG693723 | MPXV_Nig_2017_297957 | Homo sapiens | Nigeria | 2017 |
| MT903337 | MPXV-M2940_FCT | Homo sapiens | Nigeria: FCT | 2018 |
| MT903338 | MPXV-M2957_Lagos | Homo sapiens | Nigeria: Lagos State | NA |
| MT903339 | MPXV-M3021_Delta | Homo sapiens | Nigeria: Delta State | 2018 |
| MT903340 | MPXV-M5312_HM12_Rivers | Homo sapiens | Nigeria: Rivers State | 2018 |
| MN648051 | Israel_2018 | Homo sapiens | Israel | 04-Oct-2018 |
| MT903341 | MPXV-M5320_M15_Bayelsa | Homo sapiens | Nigeria: Bayelsa State | 14 – Aug- 2018 |
| MT903342 | MPXV-Singapore | Homo sapiens | Singapore | 30-Apr- 2019 |
| MT903343 | MPXV-UK_P1 | Homo sapiens | United Kingdom | 07-Sept- 2018 |
| MT903344 | MPXV-UK_P2 | Homo sapiens | United Kingdom | 11-Nov- 2018 |
| MT903345 | MPXV-UK_P3 | Homo sapiens | United Kingdom | 22-Sept- 2018 |
| MK783032 | Strain 3030 | Homo sapiens | Nigeria: Rivers State | Nov- 2017 |
| MK783030 | Strain 3025 | Homo sapiens | Nigeria: Rivers State | 30-Nov- 2017 |
| MK783029 | Strain 3029 | Homo sapiens | Nigeria: Rivers State | 06-Dec- 2017 |
| MK783028 | Strain 3019 | Homo sapiens | Nigeria: Rivers State | 09-Nov- 2017 |
| MK783031 | Strain 3020 | Homo sapiens | Nigeria: Rivers State | 09-Nov- 2017 |
| ON563414 | MPXV_USA_2022_MA001 | Homo sapiens | USA: MA | May- 2022 |
