# Supplementary material for: An APOBEC3 Mutational Signature in the Genomes of Human-Infecting Orthopoxviruses
Source: mSphere. 2023 Mar 15;8(2):e00062-23. doi: 10.1128/msphere.00062-23 (PMC10117092; doi:10.1128/msphere.00062-23)
Supplement: TABLE S3 [file msphere.00062-23-s0004.docx]

**Supplementary Table 3. List of VARV, CPXV, and FWPV strains.**

| **Accession ID** | **Strain name** | **Lineage** | **Sampling Date** |
| --- | --- | --- | --- |
| **Variola virus** |  |  |  |
| LR800244 | NA | Viking Age strain |  |
| DQ437580 | Variola virus strain Afghanistan 1970 Variolator 4 | P-I | 1970 |
| DQ437581 | Variola virus strain Bangladesh 1975 v75-550 Banu | P-I | 1975 |
| DQ437582 | Variola virus strain China Horn 1948 | P-I | 1948 |
| DQ437583 | Variola virus strain Congo 1970 | P-I | 1970 |
| DQ437584 | Variola virus strain Germany 1958 Heidelberg | P-I | 1958 |
| DQ437585 | Variola virus strain India 1964 7124 Vellore | P-I | 1964 |
| DQ437586 | Variola virus strain India 1964 7125 Vellore | P-I | 1964 |
| DQ437587 | Variola virus strain Iran 1972 2602 Tabriz | P-I | 1972 |
| DQ437588 | Variola virus strain Nepal 1973 | P-I | 1973 |
| DQ437589 | Variola virus strain Pakistan 1969 (Rafig Lahore) | P-I | 1969 |
| DQ437590 | Variola virus strain Somalia 1977 | P-I | 1977 |
| DQ437591 | Variola virus strain Sumatra 1970 V70-222 | P-I | 1970 |
| DQ437592 | Variola virus strain Syria 1972 V72-199 | P-I | 1972 |
| DQ441416 | Variola virus strain Benin, Dahomey 1968 (v68-59) | P-II | 1968 |
| DQ441417 | Variola virus strain Botswana 1972 (v72-143) | P-I | 1972 |
| DQ441418 | Variola virus strain Botswana 1973 (v73-225) | P-I | 1973 |
| DQ441419 | Variola virus strain Brazil 1966 (v66-39 Sao Paulo) | P-II | 1966 |
| DQ441420 | Variola virus strain Bangladesh 1974 (nur islam) | P-I | 1974 |
| DQ441421 | Variola virus strain Bangladesh 1974 (Shahzaman) | P-I | 1974 |
| DQ441422 | Variola virus strain Bangladesh 1974 (Solaiman) | P-I | 1974 |
| DQ441423 | Variola virus strain Congo 9 1970 (v74-227 Gispen) | P-I | 1970 |
| DQ441424 | Variola virus strain Ethiopia 1972 (Eth16 R14-1X-72 Addis) | P-I | 1972 |
| DQ441425 | Variola virus strain Ethiopia 1972 (Eth17 R14-1X-72 Addis) | P-I | 1972 |
| DQ441426 | Variola virus strain Guinea 1969 (005) | P-II | 1969 |
| DQ441427 | Variola virus strain India 1953 (Kali-Muthu-M50 Madras) | P-I | 1953 |
| DQ441428 | Variola virus strain India 1953 (New Delhi) | P-I | 1953 |
| DQ441429 | Variola virus strain Japan 1946 (Yamada MS-2(A) Tokyo) | P-I | 1946 |
| DQ441430 | Variola virus strain Japan 1951 (Harper, Masterseed) | P-I | 1951 |
| DQ441431 | Variola virus strain Japan 1951 (Stillwell, Masterseed) | P-I | 1951 |
| DQ441432 | Variola virus strain Korea 1947 (Lee, Masterseed) | P-I | 1947 |
| DQ441433 | Variola virus strain Kuwait 1967 (K1629) | P-I | 1967 |
| DQ441434 | Variola virus strain Niger 1969 | P-II | 1969 |
| DQ441435 | Variola virus strain South Africa 1965 (102 Natal, Ingwavuma) | P-I | 1965 |
| DQ441436 | Variola virus strain South Africa 1965 (103 Tvaal, Nelspruit) | P-I | 1965 |
| DQ441437 | Variola virus strain Sierra Leone 1969 (V68-258) | P-II | 1969 |
| DQ441438 | Variola virus strain Somalia 1977 (V77-1252) | P-I | 1977 |
| DQ441439 | Variola virus strain Somalia 1977 (V77-1605) | P-I | 1977 |
| DQ441440 | Variola virus strain Sudan 1947 (Juba) | P-I | 1947 |
| DQ441441 | Variola virus strain Sudan 1947 (Rumbec) | P-I | 1947 |
| DQ441442 | Variola virus strain Sumatra 1970 V70-228 | P-I | 1970 |
| DQ441443 | Variola virus strain Tanzania 1965 kembula | P-I | 1965 |
| DQ441444 | Variola virus strain United Kingdom 1946 Harvey | P-I | 1946 |
| DQ441445 | Variola virus strain United Kingdom 1946 Hinden (Middlesex) | P-I | 1946 |
| DQ441446 | Variola virus strain United Kingdom 1947 Higgins (Staffordshire) | P-I | 1947 |
| DQ441447 | Variola virus strain United Kingdom 1952 Butler | P-II | 1952 |
| DQ441448 | Variola virus strain Yugoslavia 1972 V72-164 | P-I | 1972 |
| NC_001611 | India-1967, ssp. Major | P-I | 1967 |
| Y16780 | variola minor virus complete genome | P-II | 1966 |
| **Cowpox virus** |  |  |  |
| KY463519 | CPXV_Ger1971_EP1 | Clade 1 | 1971 |
| HQ420895 | CPXV_Ger1980_EP4 | Clade 1 | 1980 |
| HQ420898 | CPXV_Ger2002_MKY | Clade 1 | 2002 |
| KC813491 | CPXV_BeaBer04_1 | Clade 1 | 2004 |
| KC813506 | CPXV_CatPot07_1 | Clade 1 | 2007 |
| KC813495 | CPXV_HumMag07_1 | Clade 1 | 2007 |
| KC813509 | CPXV_HumBer07_1 | Clade 1 | 2007 |
| KC813507 | CPXV_EleGri07_1 | Clade 1 | 2007 |
| KC813492 | CPXV_HumLan08_1 | Clade 1 | 2008 |
| KC813497 | CPXV_JagKre08_2 | Clade 1 | 2008 |
| KC813498 | CPXV_JagKre08_1 | Clade 1 | 2008 |
| KC813500 | CPXV_MonKre08_4 | Clade 1 | 2008 |
| LT896730 | CPXV_Ger2010_Racoon | Clade 1 | 2010 |
| LT896718 | CPXV_Ger2010_Alpaca | Clade 1 | 2010 |
| LT896728 | CPXV_Ger2010_Rat | Clade 1 | 2010 |
| LT896726 | CPXV_Ger2012_Alpaca | Clade 1 | 2012 |
| LT896719 | CPXV_Ger2013_Alpaca | Clade 1 | 2013 |
| LT896723 | CPXV_Ger2014_Cat1 | Clade 1 | 2014 |
| LT896725 | CPXV_Ger2014_Cat2 | Clade 1 | 2014 |
| LN879483 | CPXV_Ama_2015 | Clade 1 | 2015 |
| LT993232 | CPXV_Ger2015_Human2 | Clade 1 | 2015 |
| LT896727 | CPXV_Ger2015_Cat2 | Clade 1 | 2015 |
| LT993228 | CPXV_Ger2017_Vole | Clade 1 | 2017 |
| LT896732 | CPXV_Ger2017_Alpaca2 | Clade 1 | 2017 |
| **Fowlpox virus** |  |  |  |
| MW558073 | strain 2755 |  | 1970 |
| KX196452 | strain NX10 |  | 2010 |
| MW142017 | strain FWPV-S |  | 2012 |
| MW558068 | strain 15D039 |  | 2015 |
| MH719203 | FWPV-SD15-670.1 |  | 2015 |
| MH734528 | FWPV-SD15-670.2 |  | 2015 |
| MF766430 | 16055_trachea_170512 |  | 2016 |
| MF766431 | 16069_trachea_170323 |  | 2016 |
| MF766432 | 16117_scab_170512 |  | 2016 |
| OK558608 | FPV-CAMs |  | 2018 |
| OK558609 | FPV-COMB |  | 2018 |
| MW558072 | strain 19D064 |  | 2019 |
| MW558076 | strain V_poxine |  | 2019 |
| MW558077 | strain V_blen |  | 2019 |
| MW558078 | strain V_cmp |  | 2019 |
| MW558079 | strain V_ds |  | 2019 |
| MW558080 | strain V_kr |  | 2020 |
| MW558081 | strain V_ja |  | 2020 |
| MH709124 | FWPV-MN00.2 |  | NA |
| MH709125 | FWPV-MN00.1 |  | NA |
| AF198100 | NA |  | NA |
